# A novel causality-centrality-based method for the analysis of the impacts of air pollutants on PM_2.5_ concentrations in China

**DOI:** 10.1038/s41598-021-86304-0

**Published:** 2021-03-26

**Authors:** Bocheng Wang

**Affiliations:** Communication University of Zhejiang, Hangzhou, 310018 China

**Keywords:** Atmospheric science, Climate change

## Abstract

In this paper, we analyzed the spatial and temporal causality and graph-based centrality relationship between air pollutants and PM_2.5_ concentrations in China from 2013 to 2017. NO_2_, SO_2_, CO and O_3_ were considered the main components of pollution that affected the health of people; thus, various joint regression models were built to reveal the causal direction from these individual pollutants to PM_2.5_ concentrations. In this causal centrality analysis, Beijing was the most important area in the Jing-Jin-Ji region because of its developed economy and large population. Pollutants in Beijing and peripheral cities were studied. The results showed that NO_2_ pollutants play a vital role in the PM_2.5_ concentrations in Beijing and its surrounding areas. An obvious causality direction and betweenness centrality were observed in the northern cities compared with others, demonstrating the fact that the more developed cities were most seriously polluted. Superior performance with causal centrality characteristics in the recognition of PM_2.5_ concentrations has been achieved.

## Introduction

China is suffering from severe air pollution due to haze, especially in developed cities^1–3^. Densely populated areas such as Beijing, Tianjin, and Shanghai are often accompanied by poor air quality. Excessive emissions from the chemical industry and continuous increases in private cars lead to atmospheric photochemical pollution and high concentrations of fine particulate matter, defined as particles that are 2.5 microns or less in diameter (PM_2.5_), and other harmful substances in the air, which affect the health of people. Olmo et al.^4^ reviewed 113 studies related to atmospheric pollution and human health published between 1995 and 2009, and 109 of the analyzed studies showed evidence of adverse effects on human health. Du et al.^5^ investigated 1563 acute exacerbations of chronic obstructive pulmonary disease (AECOPD) hospitalization cases in China and analyzed the association between air pollution and these cases. Sulfur dioxide (SO_2_), nitrogen dioxide (NO_2_) and ozone (O_3_) concentrations were found to be significantly responsible for the increase in AECOPD hospitalizations. Liu et al.^6^ explored the short-term effects of air pollution on cardiovascular disease (CVD) mortality during 2013–2016. High susceptibility to air pollutants was found among females, elderly people, and ischemic heart disease patients. In particular, air pollution effects on CVD mortality were 2–8 times greater during the nonheating period than during the heating period in Northeast China. Air pollution is becoming a common concern worldwide. The improvement in air quality should be achieved by seeking the origin of pollution. Economic development has made great changes in the proportions of various components in the air^7,8^. Once the concentration of nitrogen oxides or sulfides exceeds a certain degree, it will seriously affect almost all living things on Earth. The change in air quality is a long-term and gradually accumulating process. Only limited results and incorrect conclusions will be obtained when only a certain period is considered. In recent years, the literature^9–11^ mentioned that the widespread severe haze in northern China could be blamed on the burning of straw and heating in winter. Wang et al.^12^ established twelve joint regression models by collecting four air pollutants and eight meteorological factors to analyze the impacts on PM_2.5_ concentrations and found that the haze formed in China was mainly due to NO_2_. The Chinese government also introduced policies to restrict these activities. It is well known that the burning of crop straw and rural heating are ways of living that have been handed down for thousands of years in China, while serious PM_2.5_ concentrations have not appeared until recent years. Thus, there are still many unknowns related to the formation of haze that must be studied.

Centrality-based analysis methods are widely used in many domains. Most studies calculate centrality measures such as degree, clustering coefficient or local efficiency to characterize the nodal importance based on a correlation coefficient matrix generated from a communication graph. Han et al.^13^ examined the effects of spatial polycentricity on PM_2.5_ concentrations using spatial econometric models based on a three-year panel of data for urban cities in China and used the spatial centralization index and spatial concentration index together to quantify polycentricity. Zhou et al.^14^ collected high-resolution PM_2.5_ data by mobile monitoring along different roads in Guangzhou, China, and explored the spatial–temporal heterogeneity of the relationship between the built environment and on-road PM_2.5_ during the morning and evening rush hours, calculating the betweenness centrality index for measuring the pollution impact. Despite all these studies, no research has covered further analysis with topological centrality for meteorology or air pollutants, especially in causal-based adjacent matrices. The causal direction would be such an important factor in differentiating the mutual functionality of each pollutant in the air.

Recognition of air quality by model training is a future trend in the domain of atmospheric artificial intelligence. Deep learning can be used to achieve accurate prediction with specialized knowledge. Wang et al.^15^ collected eight meteorological factors from the 100 most developed cities in China and trained an ensembled boosted tree model with 90.2% accuracy. Huang et al.^16^ developed a deep neural network model that integrated the convolutional neural network (CNN) and long short-term memory (LSTM) architectures and collected historical data such as cumulated hours of rain, cumulated wind speed and PM_2.5_ concentrations. The feasibility and practicality of the trained model were verified to improve the ability to estimate air pollution, especially in smart cites. In these studies, meteorological or pollutant factors were passed directly through machine learning models, and the intrinsic relationship among these factors was ignored during training. The spatial–temporal characteristics need to be more widely studied over a large extent.

In this paper, we studied the air pollutants NO_2_, SO_2_, carbon monoxide (CO) and O_3_ by means of time series from a large number of air monitoring data in the Jing-Jin-Ji region in China and focused on the causality influence of the accumulative process of each pollution component on air PM_2.5_. By establishing four joint regression models, we quantitatively analyzed the influence degree of air pollutants on the cause of PM_2.5_ to better clarify the formation of haze and trained a multilayer perception model to achieve improved performance compared with other methods.

## Results

**Figure 1:**
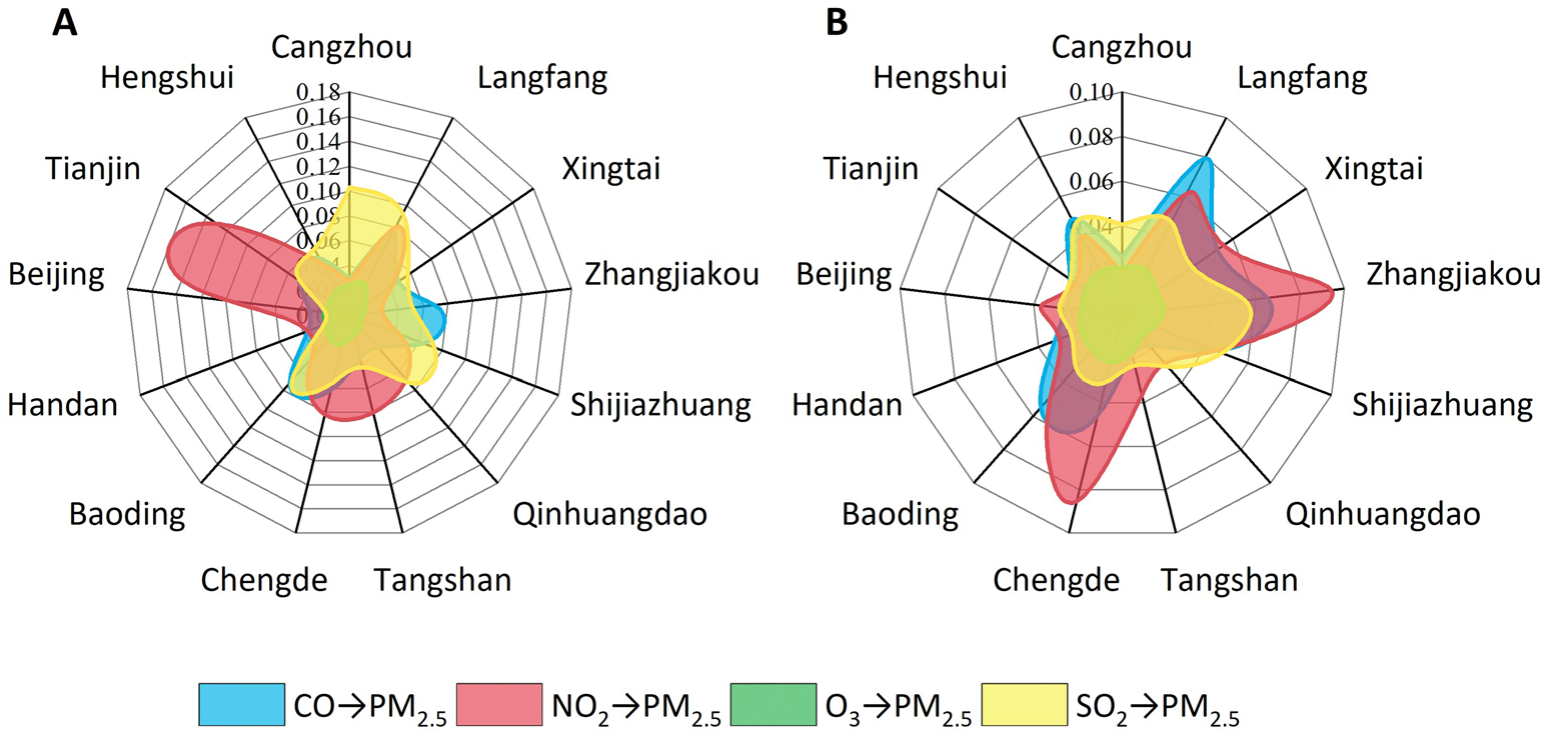
Quantitative NC impacts of (**A**) pollutants inside each city on PM_2.5_ and (**B**) pollutants from peripheral cities on PM_2.5_ in Beijing.

Figure 1 illustrates the new causality (NC) impacts from the four pollutants on PM_2.5_ concentrations. For the inner-city impact, as shown in Fig. 1A, NO_2_ has an obvious causal effect on the PM_2.5_ concentrations in Beijing and Tianjin, followed by those in Chengde and Tangshan. SO_2_ also has a significant causal effect on the PM_2.5_ concentrations in Langfang and Cangzhou. In Fig. 1B, the causality of pollutants from peripheral cities around Beijing to the Beijing PM_2.5_ concentrations is considered, and NO_2_ in Zhangjiakou and Chengde have the greatest influence, followed by CO in Langfang. SO_2_ in all the cities bordering Beijing, such as Langfang and Zhangjiakou, has certain impacts on the PM_2.5_ concentrations in Beijing. Neither O_3_ from the inner city itself that from the peripheral cities has a causal impact on the PM_2.5_ concentrations, as shown in green. Detailed information on Fig. 1 is listed in Table 1 and Table 2. The column order refers to lagging days in the NC model.

**Table 1:** NC results of the pollutants to PM_2.5_ concentrations inside each city.

	CO		NO_2_		O_3_		SO_2_	
	NC	Order	NC	Order	NC	Order	NC	Order
Cangzhou	0.030	22	0.027	11	0.025	9	0.103	22
Langfang	0.101	21	0.105	8	0.034	13	0.107	22
Xingtai	0.032	22	0.052	22	0.016	22	0.057	22
Zhangjiakou	0.085	20	0.017	10	0.013	7	0.047	13
Shijiazhuang	0.077	22	0.055	22	0.010	19	0.083	22
Qinhuangdao	0.030	10	0.074	13	0.015	13	0.089	13
Tangshan	0.035	22	0.084	8	0.016	9	0.032	22
Chengde	0.071	14	0.091	15	0.027	8	0.063	13
Baoding	0.089	16	0.064	10	0.027	10	0.091	22
Handan	0.034	22	0.010	22	0.020	22	0.023	22
Beijing	0.030	8	0.166	8	0.022	9	0.013	8
Tianjin	0.056	22	0.165	8	0.019	8	0.065	22
Hengshui	0.061	22	0.052	22	0.024	22	0.055	22

**Table 2:** NC results of the pollutants from peripheral cities to PM_2.5_ concentrations in Beijing.

City	CO		NO_2_		O_3_		SO_2_	
	NC	Order	NC	Order	NC	Order	NC	Order
Cangzhou	0.026	15	0.019	11	0.021	9	0.041	22
Langfang	0.106	17	0.082	8	0.026	9	0.056	22
Xingtai	0.038	19	0.039	8	0.018	9	0.030	22
Zhangjiakou	0.078	8	0.122	8	0.021	8	0.066	13
Shijiazhuang	0.058	22	0.050	8	0.016	8	0.052	22
Qinhuangdao	0.016	10	0.024	6	0.012	9	0.034	13
Tangshan	0.016	15	0.037	8	0.018	9	0.020	22
Chengde	0.060	13	0.109	13	0.024	8	0.035	13
Baoding	0.064	11	0.059	10	0.021	9	0.035	13
Handan	0.032	22	0.022	22	0.021	9	0.022	22
Tianjin	0.025	13	0.046	8	0.019	9	0.033	22
Hengshui	0.027	13	0.017	11	0.021	10	0.024	22

**Figure 2:**
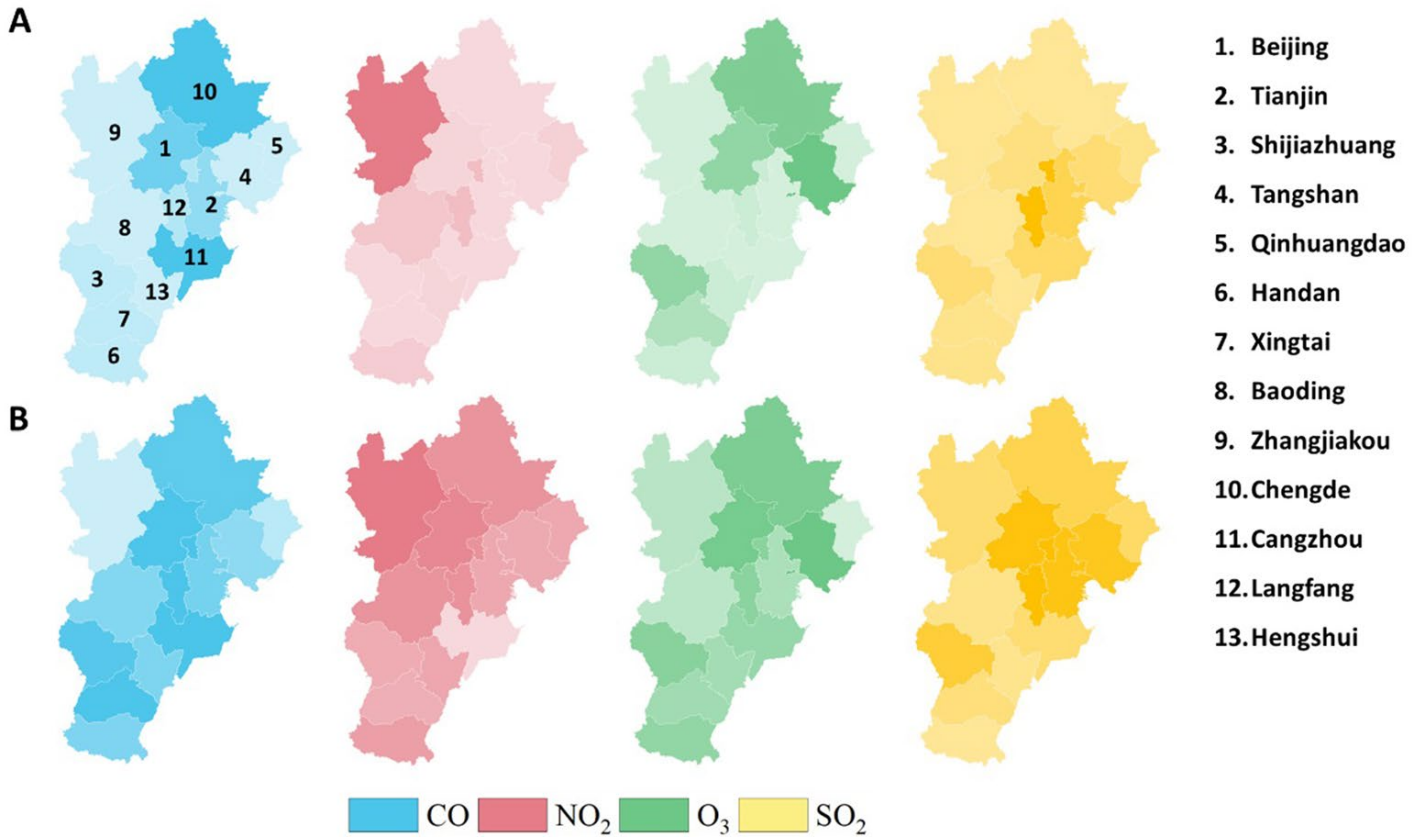
Maps of the (**A**) betweenness centrality and (**B**) clustering coefficients. The maps were drawn with R package ggplot2 version 3.3.3, https://ggplot2.tidyverse.org/.

The causality-centrality results are drawn in Fig. 2. The upper row shows the betweenness centrality under the four pollutants in the Jing-Jin-Ji region, and the bottom row shows the clustering coefficient mapping results. A large betweenness centrality is present in the northern cities, especially those adjoining Beijing, such as Chengde (CO and O_3_), Langfang (SO_2_) and Zhangjiakou (NO_2_). The discriminative ability of clustering coefficients in Fig. 2B does not behave as well as the betweenness centrality. Although the coefficient values are close to each other, it can still be inferred that pollutants around the Beijing area play an important role in the PM_2.5_ concentrations in the Jing-Jin-Ji region.

**Figure 3:**
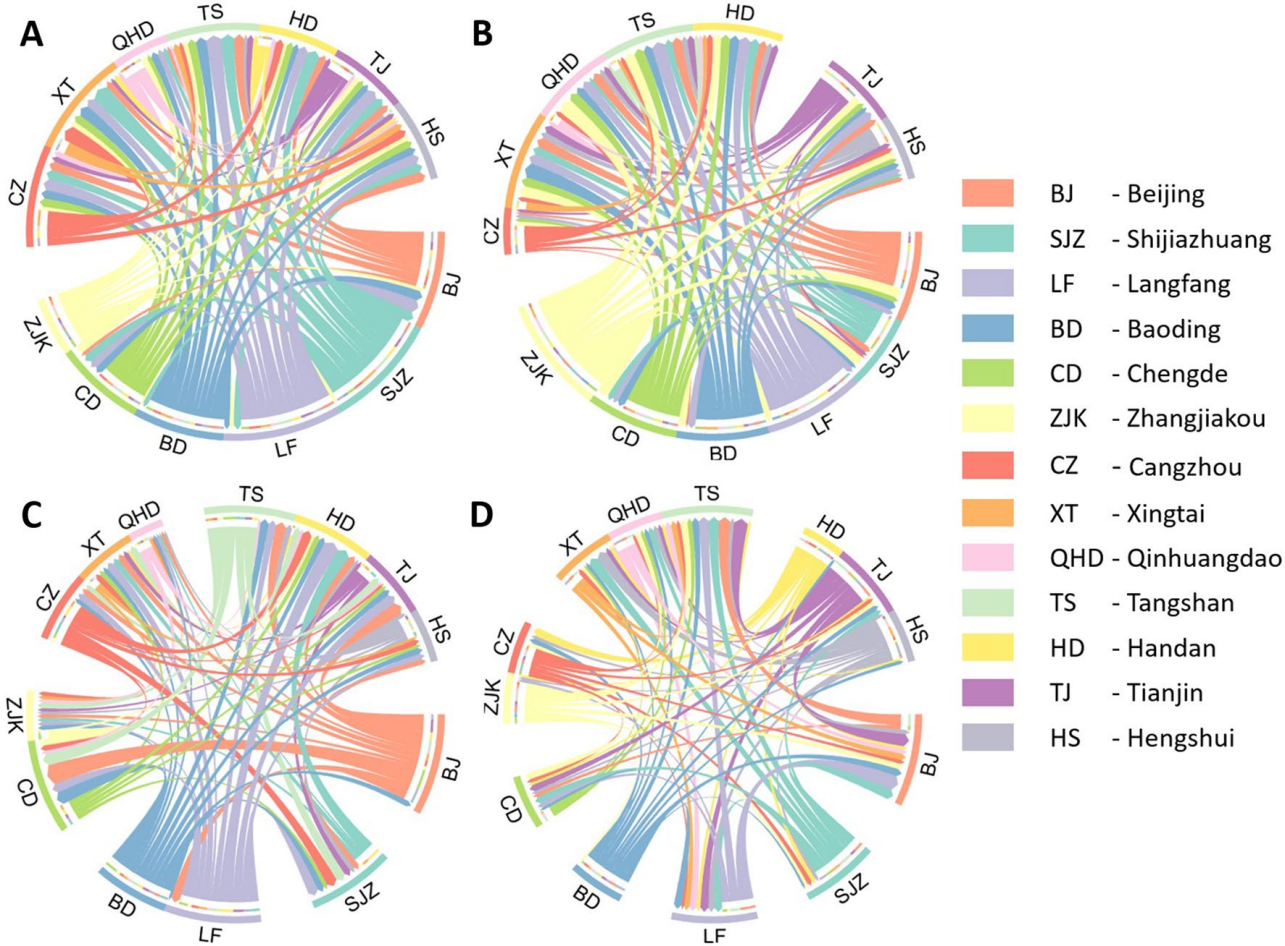
Causal direction among the cities in the Jing-Jin-Ji region. Pollutants are (**A**) CO, (**B**) NO_2_, (**C**) O_3_ and (**D**) SO_2_. Gaps are used to roughly distinguish the borders of the pollution impact directions among cities.

Figure 3 shows the causal direction among the Jing-Jin-Ji cities under the four pollutants. In Fig. 3A, the causal impacts for CO among each city are modeled by NC. The causalities in Shijiazhuang, Langfang, Baoding and most of Beijing behave as output-oriented to other cities, and input-oriented cities include Handan, Hengshui, Xingtai, Tangshan and Qinhudangdao. For the NO_2_ pollutant, in Fig. 3B, the output-oriented cities are Zhangjiakou, Langfang and most of Beijing and Baoding. Qinhuangdao, Tangshan and Handan are still input-oriented polluted cities. In Fig. 3C, obvious causal directions from Beijing, Langfang, Cangzhou and Baoding to other cities can be seen for the O_3_ pollutant. In Fig. 3D, SO_2_ in Shijiazhuang, Tianjin, Hengshui, Cangzhou, Zhangjiakou, Baoding and Handan has a direct causal impact on that in other cities, and Beijing becomes an input-oriented SO_2_ polluted city.

Table 3 lists the recognition results with causal centrality measures used in the multilayer perception (MLP) model. By constructing a three-class confusion matrix, weather was categorized into ‘Fine’, ‘Bad’, and ‘Polluted’ according to the air quality index, and the corresponding evaluation indicators, including accuracy, precision, sensitivity, and F1 score, were computed with different training parameters. The model was tested with [50, 100, 200] epochs. To accelerate the training process, the batch size was enlarged to 32 when the epoch number was 200.

**Table 3:** Model performance with causal centrality measures used in MLP.

	CO			NO2			O3			SO2		
Epoch	50	150	200	50	150	200	50	150	200	50	150	200
Batch size	16	16	32	16	16	32	16	16	32	16	16	32
Accuracy	0.7143	0.7551	0.7347	0.8163	0.9184	0.9167	0.5102	0.6122	0.5918	0.7959	0.8367	0.8163
Precision	0.7143	0.7619	0.7381	0.8333	0.9524	0.9500	0.4762	0.7143	0.7143	0.8571	0.9524	0.9048
Sensitivity	0.6522	0.6957	0.6739	0.7609	0.8696	0.8636	0.4348	0.5357	0.5172	0.7200	0.7407	0.7308
F1 score	0.6818	0.7273	0.7045	0.7955	0.9091	0.9048	0.4545	0.6122	0.6000	0.7826	0.8333	0.8085

## Discussion

In this study, the causal centrality characteristics are analyzed for the relationship between the air pollutants and PM_2.5_ concentrations of the Jing-Jin-Ji region in China. The NC-based adjacent matrices with causal direction weighting information reveal the basal functionality for the formation of PM_2.5_ under air pollutants. Different from previous studies, topological causal centrality is fully analyzed for the first time on a spatial–temporal scale. For the inner-city causal impact, NO_2_ has an obvious causal effect on the PM_2.5_ concentrations in the Beijing and Tianjin areas. The main source of NO_2_ comes from the combustion of fuel and exhaust of urban vehicles. These cities are among the most developed regions in China, and millions of vehicles are concentrated on urban roads every day^17^. Carbon monoxide emissions from heating combustion in northern China are the second leading cause of PM_2.5_ concentrations, especially in mountainous areas. None of the pollutants in Qinhuangdao have a significant causal impact on PM_2.5_ due to its special coastal narrow terrain.

### NO_2_ has the greatest impact on the PM_2.5_ concentrations in Beijing and its surrounding areas

For the pollution sources imported to the Beijing region, in Fig. 1B, NO_2_ from Chengde, Langfang and Zhangjiakou, which are located adjacent to the capital, has the greatest impact on the PM_2.5_ concentrations in Beijing. This result can be interpreted to be due to the extensive movement of the population during peak time each day^18^. Economic development in China has a great effect on air quality^19^. Although ozone has been listed as one of the air pollution observations, no significant causal direction is shown in the inner cities or peripheral cities around Beijing. According to the Spearman correlation test, ozone was negatively correlated with PM_2.5_ concentrations, which is coincident with previous studies^12,20^. Inferred from Tables 1 and 2, the closer to Beijing, the shorter the impact time of the causal function on PM_2.5_ concentrations. The lagging order in the joint regression models fluctuates around 14 (average of 16.1 in Table 1 and 13.1 in Table 2), which means that the time of the long-distance causal effect is approximately half a month.

### Northern cities have causal-central roles in the PM_2.5_ concentrations of the Jing-Jin-Ji region

Considering the causal centrality results in Fig. 2, the betweenness indicators show sensitive centrality characteristics in the cause of PM_2.5_ concentrations. For the functional topological impact from pollutants on PM_2.5_, northern cities in the Jing-Jin-Ji region have the greatest responsibility, especially those from NO_2_. These northern cities are located at the junction of the first and second ladders in China. Monsoon winds from the Inner Mongolia Plateau and Loess Plateau blow air pollutants and sand into the southern cities^21^. More importantly, remarkable differences in economic and energy consumption, development degree, and population density among these cities contribute to the uneven distribution of anthropogenic emissions^22,23^. Both natural and anthropogenic factors aggravate the PM_2.5_ concentrations.

### Causal direction showed significance in developed areas

Significant causal directions are shown in Fig. 3, especially from developed cities such as Beijing, the capital of China, and Shijiazhuang, the capital of Hebei Province. Pollutants in Beijing not only have an impact on its own region but also are responsible for pollution in other peripheral cities, as shown in Fig. 3A–C. In Fig. 3D, due to the factory relocation policy and strict emission mitigation measures in recent years, SO_2_ concentrations have decreased significantly (35.1%) in Beijing^24^, especially SO_2_ emissions in the industrial combustion and steel sectors, which decreased by 29% and 27% from 2012 to 2017^25^. This explains why Beijing acts as an import-oriented city.

### Superior performance with causal centrality characteristics in the recognition of PM_2.5_ concentrations

Previous studies^26–28^ have widely carried out research on air quality recognition mainly based on meteorological or pollutant characteristics. The centrality measured from the NC method shows superior performance in distinguishing different degrees of air pollution. The method proposed in this study can be considered efficient and practical for training the deep learning model. As shown in Table 3, the number of epochs tested ranged from 50 to 200. The best testing results were generally obtained with the parameter set (epoch = 150, batch = 16). When the epoch reached 200, nearly all critical classification indicators declined, which means that overfitting existed in the model. For all the models tested in Table 3, NO_2_ shows the most effective classification capability, which is in consensus with the results above that it has the greatest impact on the PM_2.5_ concentrations in Beijing and its surrounding areas.

## Limitations

There are some limitations in this study. First, only air pollutants are under consideration. However, air quality is affected by many factors in addition to air pollutants or meteorological factors. These factors should also be considered in the joint regression models. Second, data from restricted areas in China are collected and analyzed. Air pollution is such a complex and regional mutual weather phenomenon, and a vast spatial scale should be covered for the analysis of PM_2.5_ formation.

## Materials and method

### Materials

**Figure 4:**
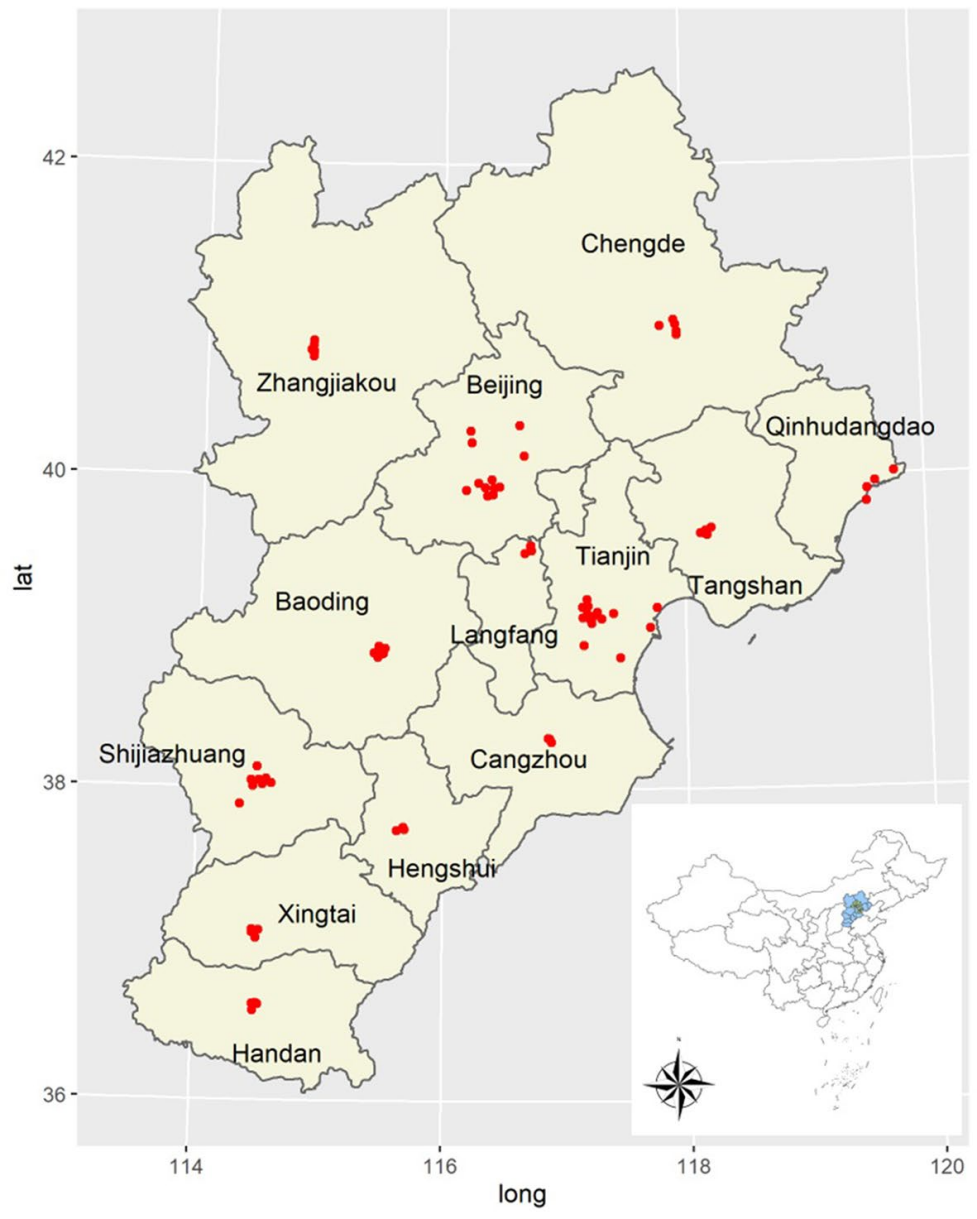
Geographical locations of air pollutant monitoring stations in the Jing-Jin-Ji region. The maps were drawn with R package ggplot2 version 3.3.3, https://ggplot2.tidyverse.org/.

Data on air pollutants were acquired from the website of the Ministry of Environmental Protection of the People`s Republic of China. This website publishes the air quality index (AQI) of each city in China on an hourly basis. PM_2.5_ (μg/m^3^), CO (mg/m^3^), NO_2_ (μg/m^3^), O_3_ (μg/m^3^) and SO_2_ (μg/m^3^) were recorded continuously at the monitoring stations. In the Jing-Jin-Ji region, 79 stations are used, which include 12 in Beijing, 15 in Tianjin, 8 in Shijiazhuang, 6 in Tangshan, 4 in Qinhuangdao, 4 in Handan, 6 in Baoding, 5 in Zhangjiakou, 5 in Chengde, 4 in Langfang, 3 in Cangzhou, 3 in Hengshui and 4 in Xingtai. The geographical locations of these stations are shown in Fig. 4. According to the occurrence of serious PM_2.5_ concentrations in China, the study period for this research was set from December 2^nd^, 2013, to February 28th, 2017.

To illustrate the interactions between pollutants and PM_2.5_ concentrations, two experiments are designed: A) the influence of local pollutants on PM_2.5_ in each city in the Jing-Jin-Ji region and B) the relationship between local PM_2.5_ concentrations in Beijing and pollutants from peripheral cities. Long-term analysis is taken into account in each experiment.

### New causality

New causality theory is derived from Granger causality (GC) theory. GC was proposed by Granger. This theory was first applied in economics and was recently widely used in neuroscience, global climate change and other scientific domains^29–31^. A brief introduction is given here. Considering a set of time series, GC exhibits the causal relationship between variations based on past values. In the form of a linear regression model, two time series are assumed to be jointly stationary. The autoregressive representations (Eq. 1) and their joint representations (Eq. 2) are described below.

1 $$\left\{\begin{array}{c}\begin{array}{c}{X}_{1,t}=\sum_{j=1}^{m}{a}_{11,j}{X}_{1,t-j}+{\epsilon }_{1,t}\end{array}\\ {X}_{2,t}=\sum_{j=1}^{m}{a}_{22,j}{X}_{2,t-j}+{\epsilon }_{2,t}\end{array}\right.$$

2 $$\left\{\begin{array}{c}{X}_{1,t}=\sum_{j=1}^{m}{a}_{11,j}{X}_{1,t-j}+\sum_{j=1}^{m}{a}_{12,j}{X}_{2,t-j}+{\eta }_{1,t}\\ {X}_{2,t}=\sum_{j=1}^{m}{a}_{21,j}{X}_{1,t-j}+\sum_{j=1}^{m}{a}_{22,j}{X}_{2,t-j}+{\eta }_{2,t}\end{array}\right.$$

where $i$ and $j$ are integer numbers ranging from 1 to the lagging order $m$ of time series $X$. ${a}_{j}$ is the coefficient of $X$. $t$ represents time. The noise terms, ${\epsilon }_{i}$ and ${\eta }_{i}$, are uncorrelated over time and have zero means. The covariance between ${\eta }_{1}$ and ${\eta }_{2}$ is defined by ${\upsigma }_{{\eta }_{1}{\eta }_{2}}$= cov (${\eta }_{1}{\eta }_{2}$). If the past values of variable ${X}_{2}$ make the estimation of ${X}_{1}$ more accurate, the noise term of ${\sigma }_{{\eta }_{1}}^{2}$ should be less than ${\sigma }_{{\epsilon }_{1}}^{2}$. In this case, ${X}_{2}$ is said to have a causal influence on ${X}_{1}$. However, if ${\sigma }_{{\epsilon }_{1}}^{2}={\sigma }_{{\eta }_{1}}^{2}$, ${X}_{2}$ has no causal impact on ${X}_{1}$. The GC value from ${X}_{2}$ to ${X}_{1}$ is therefore defined in Eq. (3).

3 $${F}_{{X}_{2}\to {X}_{1}}=ln\frac{{\sigma }_{{\epsilon }_{1}}^{2}}{{\sigma }_{{\eta }_{1}}^{2}}$$

There is no causal influence from ${X}_{2}$ to ${X}_{1}$ when ${F}_{{X}_{2}\to {X}_{1}}=0$, and if ${F}_{{X}_{2}\to {X}_{1}}>0$, ${X}_{2}$ is said to exhibit GC on ${X}_{1}$. For long-term empirical research, the vector of past values in ${X}_{1}$ or ${X}_{2}$ will be too large to build a regressive model. A general approach for determining the lagged order is the AIC-Akaike information criterion (AIC). Many algorithms can be adopted to estimate the coefficients in the joint representations. In this paper, the least squares method is used to solve the equations.

However, the value of Granger causality has been suggested to be inaccurate in some cases. It overlooks the influence of other variances in the multivariable regression model and considers only the noise terms. In 2011, Hu et al.^32^ pointed out the limitations and shortcomings of GC and provided plenty of examples that GC cannot exactly demonstrate the true causality relationship between variables. The NC method was proposed to avoid limitations and successfully applied to reveal the evident causal relationship between time series. In practice, the defined NC direction is most effective in explaining phenomena observed in nature and human activities, such as the processing of EEG signals, the increase in global temperature caused by the greenhouse effect, and the fluctuation of the stock market in the economy. In Eq. (2), past values of ${X}_{1,t-j}$ and ${X}_{2,t-j}$ occupy a large portion among the three contributors to ${X}_{1,t}$ or ${X}_{2,t}$. Based on this, a more appropriate form of causality for multivariate interactions is defined in Eq. (4).

4 $${n}_{{X}_{i}\stackrel{D}{\to }{X}_{k}}=\frac{\sum_{t=m}^{N}{(\sum_{j=1}^{m}{a}_{ki,j}{X}_{i,t-j})}^{2}}{\sum_{h=1}^{n}\sum_{t=m}^{N}{(\sum_{j=1}^{m}{a}_{kh,j}{X}_{h,t-j})}^{2}+\sum_{t=m}^{N}{\eta }_{k,t}^{2}}$$

In which, $i$ and $k$ are any unequal integers. $D$ represents the causal direction from variable ${X}_{i}$ to ${X}_{k}$. $m$ is the lagging order in ${X}_{i}$ and ${X}_{k}$. $N$ is the total length of observed time series. $n$ is the number of variables. $h$ ranges from 1 to $n$. $t$ ranges from $m$ to $N$. $j$ ranges from 1 to $m$. ${\eta }_{k,t}$ is the noise term for ${X}_{k}$ at time point $t$. In this paper, the causality relationship between pollutants and PM_2.5_ concentrations is tested, and the following model (Eq. 5) is built to describe the influence of each component contributing to haze, which appears frequently in the Jing-Jin-Ji region. Each of the four pollutants is represented by $Pollutant$.

5 $$\begin{array}{c}\underset{\{\mathit{Pollut}\mathit{ant}\}}{\mathrm{arg}\,\mathit{min} }\left\{\sum_{j=1}^{m}{a}_{11,j}{{PM}_{2.5}}_{t-j}+\sum_{j=1}^{m}{a}_{12,j}Pollutant+{\eta }_{1,t}\right\}\end{array}$$

### Graph-based centrality analysis

Graph-based centrality analysis has been a widely used method for topological relationship analysis among variables. In this study, each city in the Jing-Jin-Ji region is considered the graph node, the NC value between any two cities is regarded as the weighted edge, and an 11 × 11 square adjacent matrix is generated. Topological centrality measures, including the betweenness and clustering coefficient, are computed based on this matrix. Different from the correlation coefficient-based matrix, causality can be used to measure the causal direction between two factors. Thus, we build four-pollutant models, which correspond to four NC adjacent matrices, to analyze the causal importance from pollutants to PM_2.5_ concentrations.

The betweenness centrality is given in Eq. (6), and the clustering coefficient is defined in Eq. (7), where ${\rho }_{hj}$ is the number of shortest paths between cities $h$ and $j$, and ${\rho }_{hj}^{(i)}$ is the number of shortest paths between cities $h$ and $j$ that pass through city $i$. $N$ is the city set in the Jing-Jin-Ji region, and $n$ is the number of cities in $N$. ${a}_{ij}$ is defined as the connection weights between cities $i$ and $j$. Betweenness centrality measures the number of shortest paths that pass through a given city in a communication graph. We use this measure to characterize the importance of each city in the process of pollutant spread. The clustering coefficient can be used to measure the degree of topological clustering of pollutants around cities.

6 $${b}_{i}=\frac{1}{\left(n-1\right)(n-2)}\sum\limits_{{\begin{subarray}{*{20}c}h,j\in N\\ h\ne j,h\ne i,j\ne i\end{subarray}}}\frac{{\rho }_{hj}^{(i)}}{{\rho }_{hj}}$$

7 $$\begin{array}{c}{C}_{i}=\frac{\sum_{j,h\in N}{a}_{ij}{a}_{ih}{a}_{jh}}{\sum_{j\in N}{a}_{ij}(\sum_{j\in N}{a}_{ij}-1)}\end{array}$$

### Model training

To verify the effectiveness of the causality-centrality-based method proposed in this study, we use the calculated causality-centrality measures in MLP to determine whether these properties would bring superior classification results to the PM_2.5_ concentration prediction. MLP is a deep learning model used for classification. It mainly consists of three parts: the input layer (dependent variables), the hidden layer (interconnected neural network units) and the output layer (independent variable). The purpose of MLP is to obtain a prediction model with strong generalization ability by training the labeled input data. An MLP model with a 1024 × 1024 hidden layer is trained with these causality and centrality modalities. Instead of batch normalization, the layer normalization strategy is adopted for standardization with a range of [0, 1]. Principal component analysis is used for dimension reduction, and L_1_ embedding feature selection is implemented to avoid sparsification and overfitting. Equation (8) shows the L_1_ penalty ($\lambda$) term added to Eq. (5).

8 $$\begin{array}{c}\underset{\{\mathit{Pollutant}\}}{\mathrm{arg}\,\mathit{min} }\left\{\sum_{j=1}^{m}{a}_{11,j}{{PM}_{2.5}}_{t-j}+\sum_{j=1}^{m}{a}_{12,j}Pollutant+ \lambda |\left|Pollutant\right||+{\eta }_{1,t}\right\}\end{array}$$

After data preprocessing, the remaining causality or centrality properties are passed to the input layer of the MLP. The number of tested epochs ranges from 50 to 200, and the batch size is 16. The initial parameters of the network are set randomly, and the stochastic gradient descent algorithm is used for parameter optimization. For the output layer, the results are classified into ‘Fine’, ‘Bad’, and ‘Polluted’ after the model training and compared with the ground truth, which has been labeled before. To evaluate the performance of deep learning, indicators including the accuracy, precision, sensitivity, and F1 score are computed with different training parameters.

## Conclusion

In conclusion, this study evaluated the influence of four air pollutants on the PM_2.5_ concentrations in the Jing-Jin-Ji region with spatial and temporal comparisons by integrating the new causality and graph-based centrality analysis methods. The results indicate that NO_2_ has the greatest impact on the PM_2.5_ concentrations in the northern region of China. In addition to the pollutants exhausted inside Beijing, those from Zhangjiakou and Langfang had the greatest impact on the PM_2.5_ concentrations in Beijing. Significant causal directions are shown with significance in developed cities in China. These results imply that further work could be done for pollution control. The main source of NO_2_ resulting from human activities is the combustion of fossil fuels (coal, gas and oil), especially fuel used in cars. Therefore, higher emission standards, stricter policies for vehicle control and encouraging public transportation are expected to reduce air pollution.
